# Remote concussion history does not affect visually-guided reaching in young adult females

**DOI:** 10.2217/cnc-2019-0007

**Published:** 2019-12-05

**Authors:** Christopher Fueger, Lauren E Sergio, Sabine Heuer, Labina Petrovska, Wendy E Huddleston

**Affiliations:** 1 Department of Kinesiology: Integrative Health Care & Performance, University of Wisconsin-Milwaukee, Milwaukee, WI 53211, USA; 2 School of Kinesiology & Health Science, York University, Toronto M3J 3M4, Canada; 3 Department of Communication Sciences & Disorders, University of Wisconsin-Milwaukee, Milwaukee, WI 53211, USA

**Keywords:** cognitive load, female, long-term, motor load, mTBI, reaching, upper extremity, visual attention

## Abstract

**Aim:**

We examined the long-term effects of concussions in young adult females on visuomotor behavior during a visually-guided reaching task of various complexities.

**Materials & methods:**

20 females with a history of longer than 6 months since a concussion and 20 healthy females quickly and accurately performed a delayed reach to a previously cued target.

**Results:**

As both cognitive and motor load increased, task performance decreased for both groups (p < 0.05). However, contrary to our primary hypothesis, no differences in task performance were found between the two experimental groups (p > 0.05).

**Conclusion:**

The young adult females with a remote history of concussion demonstrated no deficits in visuomotor behavior on an attention-mediated reaching task as compared with control participants.

## Lay abstract

Current literature is inconclusive regarding the long-term effects of concussion. Some have argued that the differing results are due to many uncontrolled factors in study design. In this study, 20 females with a history of concussion more than 6 months ago and 20 healthy females performed a reaching task under different levels of difficulty. As the reaching task got harder, both groups had greater difficulty doing the task quickly and accurately (p < 0.05). Surprisingly, however, no differences in reaching performance existed between the two groups (p > 0.05). Young adult females with a remote history of concussion demonstrated no greater problems with complicated reaching tasks when compared with control participants when experimental conditions are tightly controlled.

Mild traumatic brain injuries, or concussions, have become a public health concern of large magnitude [1]. An estimated, 1.6 million to 1.9 million sports-related concussions occur annually in the USA in athletes under the age of 18 [2]; however, the actual incidence of concussions may be higher due do athletes not reporting their injury [3,4]. While awareness for the prompt identification and appropriate immediate treatments for concussions continues to advance, a concurrent need exists to understand the long-term effects of concussions, especially for individuals with a remote history of concussion. For the purposes of this study, remote will refer to individuals whose most recent concussion was sustained at least 6 months prior to study participation and have returned to normal activities. Experimental assessment of the long-term effects of concussions on individuals usually falls into one of two categories: cognitive function testing or motor function testing.

Neuropsychological investigations of cognitive function in individuals who have sustained concussions revealed overall cognitive decline [5,6], poorer verbal memory [7–9], longer processing speed [10–12], poorer visuospatial memory [7] and attentional deficits [10,13]. Motor deficits identified in previously concussed individuals found decreased postural stability and bradykinesia [14], reduced upper limb movement accuracy, slowed ballistic velocity [15] and increased choice reaction times [16]. Although independently assessing cognitive and motor functioning has its merits, a gap exists that may prevent researchers from fully detecting behavioral deficits. Cognitive and motor systems can function independently, but also interact with each other using finite attentional resources needed to complete a task [17,18]. However, traditional methods of testing individuals who have sustained a concussion often fall short of sufficiently challenging the participants to their attentional capacity limits, limiting the detection of behavioral deficits. Therefore, a need exists to examine this cognitive–motor interaction with a task that sufficiently challenges previously concussed individuals to the limit of their capacity of attentional resources.

Every day, vision guides one’s actions to help one successfully navigate through a complex environment. Visuomotor behavior can be exemplified with tasks as simple as reaching for a pot on the stove to remove it before the pot boils over; to walking down a sidewalk while talking to a friend, yet still avoiding any cracks or potholes as to avoid a fall; or more complex behavior such as when driving a car during rush hour traffic and taking corrective actions to avoid a motor vehicle accident. When our visual and motor systems interact efficiently, we may not fully appreciate how beneficial flawless visuomotor behavior can be to daily functioning. However, when damage to the brain occurs, deficits in visuomotor behavior can arise. These deficits can have adverse effects on day-to-day activities, which may lead to a decreased quality of life. For example, stroke survivors have shown deficits in visuomotor behavior (i.e., visual attention) that were significantly correlated with increased fall rates and decreased capability to perform activities of daily living [19]. Also, patients diagnosed with Alzheimer’s disease have shown impaired visuomotor integration correlated to cognitive decline that has implications for successfully completing more complex visuomotor tasks, such as ascending a flight of stairs [20].

Visuomotor behavior has also been investigated in individuals who have sustained one or more concussions, although the results have been equivocal. Visuomotor behavioral deficits, when compared with healthy controls, include: slower reaction times in a reverse choice lower extremity stepping task [21], impaired upper limb visuomotor performance on a 1D tracking task [15,22], decreased visuomotor processing speed on Trail Making Test A and B [12], decreased obstacle avoidance while performing a secondary attention task [23] and altered gait kinematics during a varying obstacle avoidance task [24]. Conversely, others have found no difference in reaction times on upper extremity pointing task [21] and no differences in reaction times during a pointing task with varied target size [25].

Potential factors leading to the inconsistent results reported above include variation in time since injury [16,25], age [13,26] and sex [25,26] of the participants, as well as the number of previous concussions [8,9,14,25,27–29]. Additionally, even though cognitive demand has varied in some experiments of visuomotor behavior in previously concussed individuals, questions have arisen as to whether enough cognitive load was used to truly tax the participants’ abilities [25]; perhaps the experimental tasks have not sufficiently challenged the participants and actual behavioral deficits have been masked in the findings.

The purpose of this study was to examine the long-term effects of a history of concussions in young adult females on visuomotor behavior during a visually-guided reaching task of various complexities. A behavioral task was designed that varied both cognitive and motor demand of the task, as well as careful control for confounding factors of time since injury, age and sex of the participants. We hypothesized that both temporal and movement performance related to a targeted reaching task would decline based on cognitive and motor load (to varying degrees based on the task condition), and that this decline would be accentuated in participants with a remote history of concussion who potentially had greater limitations in attentional resources than healthy adults. However, even when controlling for number of concussions and a number of other factors, female participants with a remote history of concussion performed similarly to the control group.

## Materials & methods

### Participants

41 healthy young adult females, aged 18–28 years, with normal or corrected-to-normal vision and either no history of concussion or at least one self-reported concussion at least 6 months ago were recruited. Due to technical difficulties during data collection, the data from one participant was corrupted and was not included in the final analysis. 20 females without a history of concussion (CONTROL age: 21.2 ± 2.16 years, left-handed n = 1) and 20 females with a history of concussion (CONC age: 22.3 ± 2.43 years, left-handed n = 1) participated in the study. We chose to include individuals with a remote history of concussion (>6 months since last concussion) who were asymptomatic due to conflicting information regarding the long-term effects of concussion because of methodological issues such as experimental design or the heterogeneity of participants. These factors were directly addressed in the design of the current study. Males were excluded for two reasons. First, a difference exists between sexes when using cognitive tests to evaluate individuals with a history of concussions [7,26]. Second, females represent an understudied population in the concussion literature, thus warranting a need for increased investigation [30,31]. Additional exclusion criteria included: a concussion within the previous 6 months, an inability to sit comfortably for up to an hour and a half, self-reported neurological deficits other than a concussion, a diagnosed learning disorder, a diagnosis of ADD/ADHD or dyslexia, a current psychological disorder which requires medication (other than a mood disorder), any vision impairment that would preclude seeing the stimulus and an arm or spinal cord injury in the last 6 months. A power analysis conducted using G*Power (Duesseldorf, Germany) software [32] confirmed at least 18 participants per group were required to achieve adequate power. Informed consent, as approved by the Institutional Review Board, was obtained from the potential participants (IRB# 15.260). All participants received compensation upon completion of the study. Additionally, all experimental data are available upon request from the corresponding author.

### Procedure

Participants were asked to temporarily cease caffeine intake at least 2 h before their testing session to mitigate the acute effects of caffeine on cognitive function or reaction times [33,34]. After obtaining informed consent, participants’ vision was screened using a Snellen eye chart to check near visual acuity [35]. Participants completed a Concussion History and Symptom Survey. Section 1 (Concussion History) obtained participants’ previous history of diagnosed (by a medical provider) and undiagnosed concussions. The purpose of this section was threefold. First, participants recorded the length of time from their most recent concussion, if applicable. Second, participants verified they had been medically cleared from the most recent concussion. Third, both diagnosed and undiagnosed concussion history was documented, therefore assessing the actual number of previous concussions. Since the intake of total concussion history was obtained via self-report, the number of diagnosed and undiagnosed concussions was summed to arrive at a total number of previous concussions for each participant. Section 2 (Concussion Symptom History and Evaluation) served two purposes. First, the Symptom History checklist recorded symptoms from other head injuries not diagnosed as a concussion. LaBotz *et al.* [36] developed this Concussion Symptom survey as a more sensitive measurement of previous concussion history, thus it was included to fully capture the number of previous concussions. The second checklist, Symptom Evaluation, was from the Sport Concussion Assessment Tool 3 (SCAT3) [37]. The purpose of this checklist was to verify that participants were asymptomatic by current standards. The Concussion History and Symptom Survey classified participants into one of two experimental groups: no concussion history (CONTROL) and concussion history (CONC).

A Participant Lifestyle Questionnaire assessed lifestyles that might affect how participants performed on this task. These included sleep patterns, nicotine use, caffeine use and prescription medication for mood disorders, which all have been supported in the literature to alter cognitive function or reaction times [33,38–41]. Participants also answered a Sports Participation Questionnaire to assess the frequency, type and competitive level of any sports played by the participants [42].

Participants completed the Edinburgh Handedness Inventory to assess hand dominance [43]. Scores on this inventory determined the hand used during the experimental trials. The participants’ dominant arm (from shoulder to the tip of the index finger) was measured. This measurement was used to compare to the theoretical path lengths so that the maximal path lengths would be less than 80% of the total arm length. This comparison ensured no participant performed reaches with maximal extension of the arm, which could have led to fatigue and influenced the dependent measures.

A MiniBird kinematic system (Ascension Technology, VT, USA) was used to collect movement data during the experiment. A customized LabView program (BloomTech, WI, USA) was used to calculate the variables of interest, sampling the analog data at 111 Hz. The sensor was secured to the participants’ dominant dorsal-side index finger with medical tape. The same customized LabView program presented the visual stimulus for the reaching tasks on a laptop computer. The laptop computer was connected to a LCD projector that displayed the reaching task on a custom-built back-projection screen.

**Figure 1.**
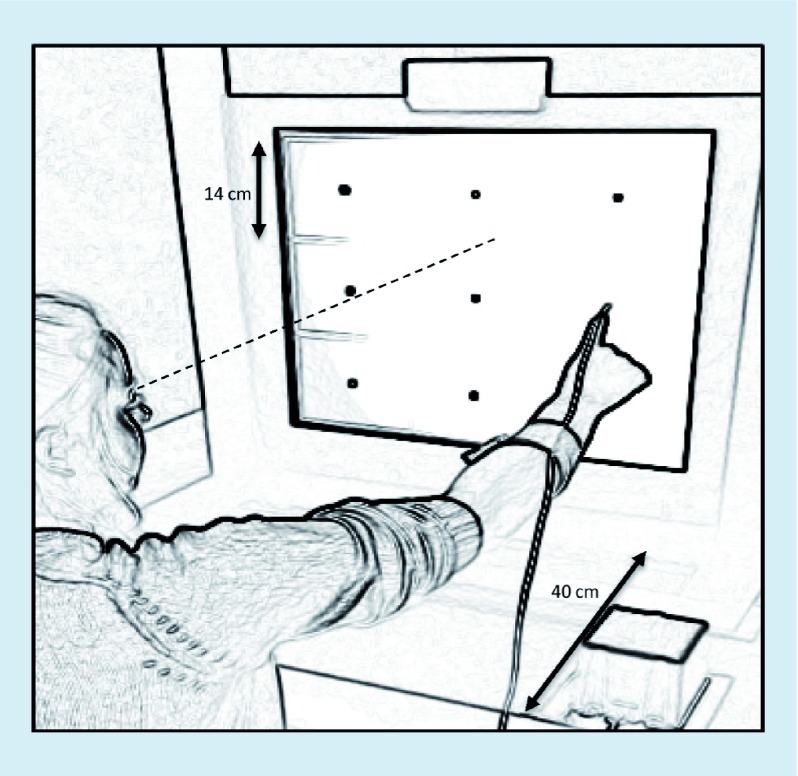
Experimental set-up. Each 14 cm^2^ had a central 1 cm^2^ target. The dashed line indicates the alignment of the stimulus display relative to the participant’s eye position.

Participants sat upright on a stationary chair in front of the projection screen (Figure 1). The projection screen displayed a 3 × 3 grid of white squares with a central target. Each participant completed a calibration process to determine the location of each of the eight targets and the home position in 3D space.

**Figure 2.**
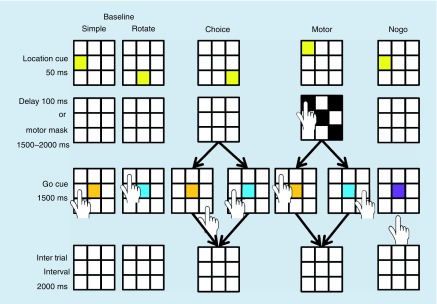
Trial sequence for the three visually-guided reaching task conditions. The overall task was for participants to touch a square based on task instructions. Above are examples of each task condition. In the BASELINE-SIMPLE task (orange go cue), participants touched the previously cued square after a brief delay. In the BASELINE-ROTATE (blue go cue), participants mentally rotated the target location clockwise three spaces from the original cue location. The CHOICE condition, participants applied either the SIMPLE rule or the ROTATE rule based on go cue color. The MOTOR condition required participants to touch the four black squares in a self-selected order during the delay before touching the appropriate square based on task rule. Catch trials (purple go cue) required participants to inhibit movement during the go cue and occurred occasionally (10% of total trials) in each of the task conditions.

Participants performed each of two trial types under three different conditions (Figure 2). In the first trial type, participants touched the center of the cued peripheral square after a brief delay (SIMPLE). In the second trial type, participants touched the center of the square three spaces in the clockwise direction from the original location cue, again after a brief delay (mental rotation; ROTATE). The cued target location (the eight peripheral squares) in both trial types was indicated by the target square briefly turning yellow. The go cue was always presented in the center square and the trial type was indicated by the color of the cue (orange – SIMPLE; blue – ROTATE). If the go cue was purple (NOGO), participants withheld their reach as a catch trial. Zero to three catch trials occurred in every block of 16 trials for all of the task conditions described below such that catch trials represented 10% of the total trials in a task condition. Due to the low number of NOGO trials, these data were excluded from subsequent analysis.

Participants performed these two types of trials under three task conditions (Figure 2). During the BASELINE condition, participants completed entire blocks of either SIMPLE trials or ROTATE trials. The SIMPLE BASELINE was the easiest condition in terms of overall cognitive demand. The ROTATE BASELINE condition added a level of cognitive complexity to the task as participants had to mentally rotate the target location (Figure 2). During the CHOICE reaching condition, another level of cognitive demand was added (Figure 2). The target cue was presented as before, but the participants did not know if the trial was to be SIMPLE or ROTATE until the go cue was presented, thus requiring participants to quickly identify the color of the go cue, apply the associated rule and make an accurate reach to the correct square. Also, the number of potential motor trajectories increased from one to two in this condition, increasing motor load.

The third condition (MOTOR) was the same sequence as the CHOICE Condition with the addition of a second motor task over the delay period. To provide sufficient time for participants to complete the second motor task, the delay was lengthened to 1500–2000 ms when four black squares randomly appeared (Figure 2). Participants touched all four black squares in a self-selected order and returned to the home position as quickly as possible to wait for the go cue for the originally targeted position. The motor mask was randomly assigned to locations on the grid and the location of one of the four black squares was counterbalanced between being congruent and incongruent to the original location cue. This condition added motor interference and thus increased the motor load, to the task condition as participants would potentially have difficulty preplanning their reach to the originally cued target location. For this study, motor load was defined as the amount of motor planning and execution required to perform the reaching task. We increased motor load by increasing the number of possible movement trajectories in the CHOICE and MOTOR conditions, and additionally by adding a motor ‘mask’ in the MOTOR task.

Due to the nature of the task and to limit learning bias, the following assignment of task conditions was used. The SIMPLE BASELINE and ROTATE BASELINE conditions were counterbalanced among participants and were the first two conditions participants performed. The CHOICE and MOTOR conditions were also counterbalanced between the two conditions, and always followed the two baseline conditions. Participants received practice trials before each new task condition and obtained >70% target selection accuracy to proceed to the corresponding experimental task condition. Of the additional practice trials needed to obtain >70% target accuracy, only one participant (CONTROL, MOTOR) needed additional trials due to not achieving the minimum accuracy threshold. All other additional trials were due to timing errors (either participants initiated movement before the go cue was presented or did not return to the home location within the duration of time allocated for the motor mask). Participants also received rest breaks as needed to prevent fatigue.

### Analysis

Independent variables were trial type (SIMPLE and ROTATE), task condition (BASELINE, CHOICE, MOTOR) and concussion history (CONTROL, CONC). Dependent variables were target selection accuracy, reaction time, reaction time variability, movement time, movement time variability, path length, path length variability, end-point accuracy and end-point variability (horizontal and vertical). A mixed 2 × 3 × 2 repeated measures analysis of variance (ANOVA) was run separately for each dependent measure using SPSS software (v. 22 IBM, NY, USA). We performed a Shapiro–Wilk test of normality on all dependent measures. End-point accuracy, normalized path length and selection accuracy were not normally distributed and thus a Huynh–Feldt correction was used and reported for these measures. Significance level was set at α = 0.05 with adjustments made for multiple comparisons using the Holm–Bonferroni method [44].

We performed one-tail t-tests when a directional hypothesis was tested (e.g., symptom history and severity between healthy and concussed participants and the main effects of task condition and group assignment). Paired t-tests were used for tests of differences among task conditions while unpaired t-tests were used for any tests between the two participant groups.

The customized LabView program calculated the variables of interest. Target selection accuracy was a percentage of reaches to correct target locations. Target selection was based on the participant’s index fingertip getting within a ‘target ellipse’ measuring 6.0 cm in the X and Y directions and 1.0 cm in the Z direction of the target. Reaction time was the elapsed time from the presentation of the go cue to the initial movement of the participant’s index finger as calculated by the time the finger moved more than 2.0 cm in any direction (‘home base ellipse’) from the home base after the ‘go’ cue presentation. Movement time was measured as the elapsed time from the initial movement of the participants’ finger until a ‘target ellipse’ was reached. The path length variable was a normalized value by dividing the actual path length as measured by the shortest 3D distance between the home base and the target location. The formula for each participant’s path length was:

(Eq. 1) $$\text{P}\text{A}\text{T}\text{H}=\sum_{\text{i}=1}^{\text{n}}\sqrt{{\left({\text{x}}_{\text{l}}-{\text{x}}_{\text{k}}\right)}^{2}+{\left({\text{y}}_{\text{l}}-{\text{y}}_{\text{k}}\right)}^{2}+{\left({\text{z}}_{\text{l}}-{\text{z}}_{\text{k}}\right)}^{2}}$$

Where for each data sample ‘l’, the difference was calculated for each dimension relative to the position at the previous time point ‘k’ and summed for the entire trajectory (n = final time point in the series) from home base to the target.

The formula for normalized path length was:

(Eq. 2) $$\text{P}\text{A}\text{T}{\text{H}}_{\text{n}\text{o}\text{r}\text{m}}=\frac{\text{P}\text{A}\text{T}{\text{H}}_{\exp}}{\text{P}\text{A}\text{T}{\text{H}}_{\text{a}\text{c}\text{t}\text{u}\text{a}\text{l}}}$$

Reaction time variability, movement time variability and path length variability were all calculated as the respective within-subject coefficient of variation. End-point accuracy was divided into horizontal and vertical components of absolute error (z-plane was fixed as the participants were touching a 2D screen) and defined as the distance from the calibrated target location to the experimental touch of the target. From the end-point accuracy values, coefficients of variation were calculated for both the horizontal and vertical directions, thus producing end-point variability in either direction.

For the final analysis, trials were excluded based on four criteria. First, trials with reaction times (RT) less than 150 ms and greater than 2000 ms were excluded from the final analysis (RT error). Second, a ‘miss’ error occurred if the participant failed to initiate movement after a valid go cue typically because the participant missed the location cue. In this case, the entire trial was excluded from the final analysis. Third, a ‘no touch’ error occurred if the participant’s touch did not register due to the MiniBird sensor failing to pass the boundaries of the target ellipse. Trials classified as ‘no touch’ errors were excluded from the final analysis. Fourth, if a participant touched the incorrect target location, only the dependent variable ‘target selection accuracy’ was included from that trial in the final analysis.

## Results

### Concussion history & symptoms

The concussion group averaged 3.0 ± 1.6 concussions (*R* = 1 – 6 concussions) and the time since the most recent concussion was 33.24 ± 36.12 months (*R* = 6 – 156 months). Of the 60 total concussions reported, 33 (55%) were diagnosed by a medical practitioner (MD, ATC, PT, RN). An evaluation of previous head injuries and the resulting symptoms identified four participants from the CONTROL group who may have sustained an unreported concussion. Data from the control subjects were analyzed with and without these four participants separately (See Results: Additional Analyses). As expected, SCAT3 total symptoms scores (CONTROL 0.3 ± 0.91; CONC 3.6 ± 5.28) significantly differed between groups (t _(38)_ = −2.80, 1-tail; p < 0.05) as well as total severity scores (CONTROL 0.5 ± 1.79; CONC 6.0 ± 10.98; t _(38)_ = −2.21, 1-tail; p < 0.05). Concussion characteristics of the participants with a history of concussion are displayed in Table 1.

**Table 1.** Summary of the concussion history and symptom survey of participants with a history of concussion.

Participant	Age (years)	Self-reported concussions (n)	Time since last concussion (months)	SCAT3 symptom score (22)	SCAT3 severity score (132)
1	20	3	30	0	0
2	27	1	156	4	7
3	22	3	75	0	0
4	21	4	60	0	0
5	21	5	11	13	24
6	23	2	6	4	4
7	24	6	24	0	0
8	23	4	6	8	11
9	28	5	12	20	45
10	19	1	54	0	0
11	19	4	14	2	2
12	23	4	8	0	0
13	22	1	9	2	2
14	23	2	7	5	6
15	19	3	28	0	0
16	22	3	24	0	0
17	20	1	24	8	12
18	24	1	24	5	5
19	22	5	24	0	0
20	24	2	72	1	1
Mean ± SD	22.3 ± 2.43	3.0 ± 1.6	33.24 ± 36.12	3.6 ± 5.28	6.0 ± 10.98

SCAT3: Sport Concussion Assessment Tool 3; SD: Standard deviation.

### Participant Lifestyles

All participants reported as nonsmokers/nicotine users and all participants refrained from the intake of caffeine at least 2 h before their scheduled testing session. Participants reported their fortnightly average amount of sleep (CONTROL 6.7 ± 0.86 h; CONC 6.6 ± 1.01 h) and the total hours of sleep from the night before their experimental session (CONTROL 6.6 ± 1.09 h; CONC 6.9 ± 1.45 h). A one-way ANOVA (F _(3, 76)_ = 0.22; p > 0.05) revealed no difference in hours of sleep between the two groups, nor any differences between the hours of sleep from the night before compared with the fortnightly average. Additionally, we collected data on prescription medication usage for mood disorders across the CONTROL group (n = 1; Effexor: 75 mg/daily) and the CONC group (n = 4; Sertraline: 50 mg/daily [n = 2]; Fluoxetine: 40 mg/daily; unspecified thyroid medication: 75 mg/daily). All participants who reported prescription medication for mood disorders were on their respective medication for at least 6 months (range: 0.5–5.0 years) and had taken their respective medication before the testing session.

### Current sports participation

Tegner Activity Level Scale [42] scores (out of a possible score of ten) were collected for both groups. An independent t-test (t _(38)_ = −2.67, 2-tail; p < 0.05) revealed a significant difference between the two groups (CONTROL 5.6 ± 1.70; CONC 7.2 ± 2.07).

### Visually-guided reaching task

**Figure 3.**
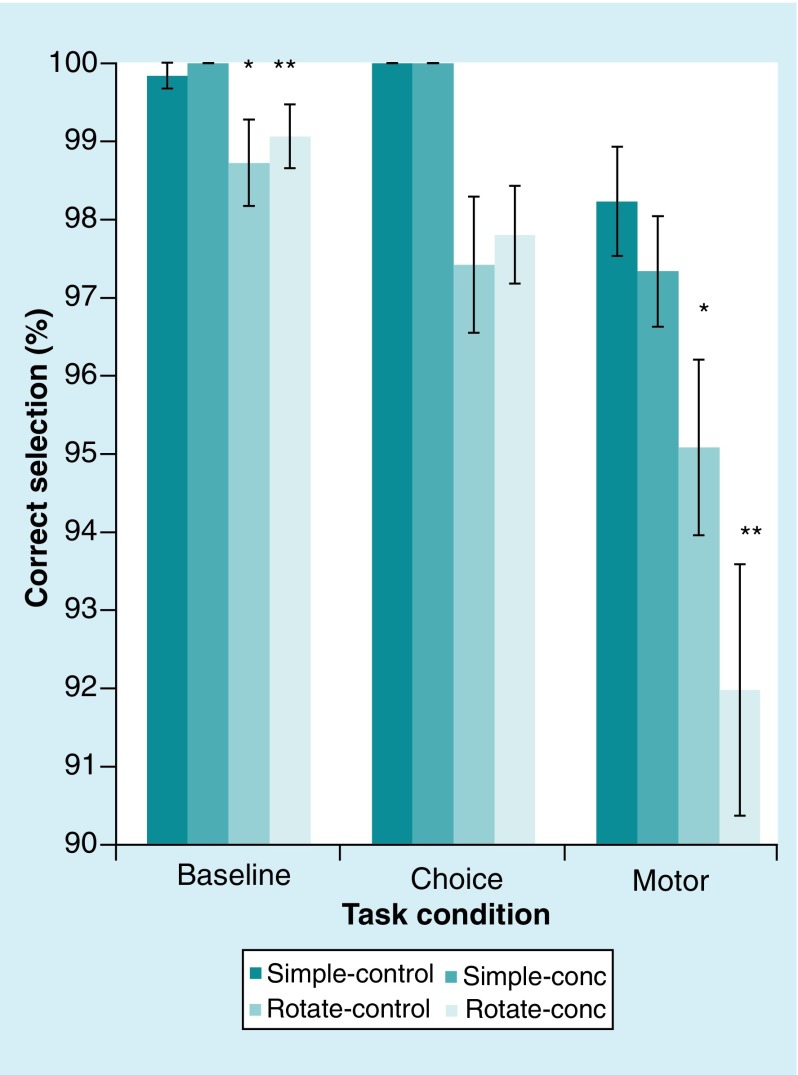
Target section accuracy. Both main effects of trial type (p < 0.05) and task condition (p < 0.05) were significant as well as the interaction between trial type and task condition (p < 0.05). *, **p < 0.05. Error bars represent standard error of the mean.

The lowest Target Selection Accuracy (Figure 3) was 91.98% (CONC, ROTATE trial type, MOTOR task condition) with the remaining accuracies ranging from 95.08 to 100.00%. Given the *a priori* prediction that the poorest performance on the visually-guided reaching task would occur during the largest cognitive and motor demand, further analysis was conducted. Paired t-tests revealed a significant difference in the ROTATE trial type between the BASELINE and MOTOR condition for target selection accuracy within both the CONC group (t _(19)_ = 4.32, 1-tail; p < 0.001; BASELINE 99.06% ± 1.79; MOTOR 91.98% ± 7.02) and the CONTROL group (t _(19)_ = 3.26, 1-tail; p < 0.005; BASELINE 98.72% ± 2.41; MOTOR 95.08% ± 4.91). However, an independent t-test (t _(38)_ = 1.62, 1-tail; p = 0.054) revealed no significant difference in the MOTOR condition for the ROTATE trial type for target selection accuracy between the two groups (CONTROL 95.08% ± 4.91; CONC 91.98% ± 7.02).

**Figure 4.**
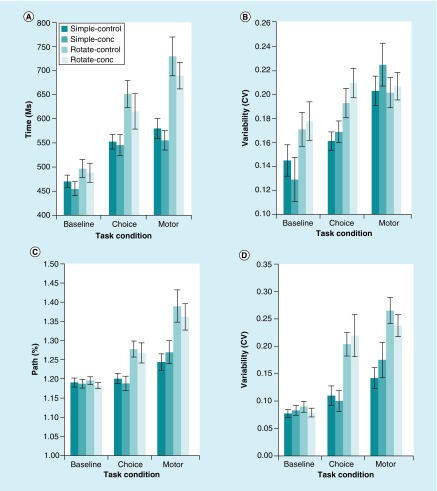
Examples of temporal and kinematic variables. **(A)** Average reaction time. **(B)** Reaction time variability. **(C)** Path length. **(D)** Path length variability. For all four graphs illustrated, both main effects of trial type (p < 0.05) and task condition (p < 0.05) were significant as well as the interaction between trial type and task condition (p < 0.05). However, no group interactions were revealed (p > 0.05). Error bars represent standard error of the meann.

Results of the repeated measures ANOVAs for the other dependent measures revealed significant main effects for trial type (p < 0.05) across the dependent measures of target selection accuracy (Figure 3), reaction time (Figure 4A), reaction time variability (Figure 4B), movement time, movement time variability, path length (Figure 4C) and path length variability (Figure 4D). Significant main effect for task condition (p < 0.05) was found for all dependent measures except horizontal end-point variability (p > 0.05). Significant interactions of Trial Type X Task Condition (p < 0.05) were found for target selection accuracy (Figure 3), reaction time (Figure 4A), reaction time variability (Figure 4B), movement time, path length (Figure 4C) and path length variability (Figure 4D). No significant interactions were revealed for the main effect of group membership (p > 0.05) or the interactions of Trial Type X Group (p > 0.05), Task Condition X Group (p > 0.05) and Trial Type X Task Condition X Group (p > 0.05).

### Additional analyses

To confirm that a Type II error was not committed relative to the between-subjects factor of concussion history, various analyses were conducted to address potential pitfalls to the experimental design. Each analysis was performed independent of the other analyses.

First, to control for the differences revealed from the Tegner Activity Level Scale, participants in the CONTROL group with a score of 5 or lower were removed (n = 8), leaving 12 participants in the control group. An independent t-test (t _(30)_ = −0.95, 2-tail; p > 0.05) revealed no longer any difference between the two groups in activity level with this change in group membership (CONTROL 6.6 ± 1.08; CONC 7.2 ± 2.07). However, after this adjustment, still no significant differences were found between the previously concussed group and the controls in any dependent measure.

Next, the four participants in the CONTROL group with a suspected but unreported concussion were removed from the CONTROL group (leaving n = 16) and the analyses were repeated. No significant differences were found between the CONC and CONTROL groups for any dependent measure.

Third, three participants in the CONC group self-reported a history of concussion; however, all of their concussions were classified as undiagnosed (by a medical practitioner). To adjust for any influence on the dependent measures due to misreporting a concussion, the three individuals were removed from the CONC group (leaving n = 17) and the analyses were repeated. After this adjustment, no significant differences were revealed between the CONC and CONTROL groups for any dependent measure.

Fourth, previous evidence suggests that, if an individual has a history of three or more concussions, they demonstrate poorer performance on a cognitive task when compared with both individuals with one or two previous concussions and control participants [6,7,11]. In the previous analysis we had collapsed all people with a concussion into the same group. For the next analysis, the CONC group was divided into two groups based on concussion history (one or two [n = 8] vs three or more previous concussions [n = 12]). An independent t-test (t _(18)_ = −0.13, 2-tail; p > 0.05) revealed no difference on the Tegner Activity Level Scale between the two new CONC groups with this change in group membership (CONC_1-2_ 7.1 ± 2.16; CONC_3+_ 7.3 ± 2.09). After this group reassignment, no significant differences were found among the three groups for any dependent measure.

Fifth, to adjust for the effects of prescription medication for mood disorders on the dependent measures [33,40,41], the five participants (CONTROL n = 1) were removed from their respective groups. The analyses were performed and no significant differences existed between the CONTROL and CONC groups for any of the dependent measures.

At last, the possibility existed that an individual was inherently better at the visually-guided reaching task regardless of their concussion history (or lack thereof). To control for this potential confound, two separate analyses were conducted:

- Individual difference scores for both trial types (SIMPLE and ROTATE) were calculated using the respective task condition statistics of: choice – baseline, motor – baseline and motor – choice;
- Individual normalized scores were calculated for both trial types by dividing the task condition statistics of choice and motor by its respective baseline value.

The results of the subsequent analyses were consistent with the original results of no significant differences between control participants and those with a history of concussion. Based on the multiple analyses, and the consistent finding of no significant differences between the two experimental groups, the likelihood of a Type II error was rejected.

## Discussion

We examined the long-term effects of concussions in young adult females on visuomotor behavior during a visually-guided reaching task of various complexities by designing a behavioral task that varied both cognitive and motor demand of the task to overextend attentional resources, as well as carefully control for confounding factors of time since injury, age and sex of the participants. Novel to this body of work was the introduction of motor interference, which we altered by increasing the number possible motor responses [45] or by introducing motor interference to the task [46]. We also increased cognitive and motor load by combining the two elements in the MOTOR condition to further challenge participants. As cognitive and motor demand increased, both temporal and kinematic characteristics decreased for both experimental groups. This is important as a sufficient increase of the cognitive load of a task is required to elicit a detectable difference between individuals with and without a history of concussion [16,25], which was demonstrated in the present study since both groups showed a decline in performance as load increased. However, no significant differences existed between the individuals without a history of concussion and those with a long-term history of concussion for any condition. Within-subject variability is considered a measure of the efficiency of the allocation of attentional resources as participants are challenged by increases in cognitive load [47]. No significant differences in within-subject variability were detected between the two groups in any of our results. Given these findings, attentional resources did not appear to be impaired in this population at least 6 months after the most recent concussion.

Our results add to the already divergent results of the effects of a remote concussion on visually-guided reaching. These current findings agree with those reported by Locklin *et al.* [25] that reported no significant differences in task performance between the two experimental groups, although our task was more difficult as we changed the reaching target on each trial. However, the results of the current study disagree with those reported by Brown *et al.* [16]. Brown *et al.* [16] reported a significant difference in reaction time, movement time and end-point variability between the group with a history of concussion and those with no pervious concussions. One possible explanation for the conflicting results is the time since most recent injury of the participants. In the current study, all participants were asymptomatic by current standards and at least 6 months removed from their most recent concussion. However, Brown *et al.* [16] included eight of the 18 previously concussed participants who were asymptomatic by current standards yet within only 1 month from their most recent concussion. Given the detrimental effects of concussions on cognitive performance during the acute phase of recovery [31,48–50], the possibility of this factor influencing the results reported by Brown *et al.* [16] exists.

When using behavioral measures to detect differences between asymptomatic individuals with a remote history of concussion and controls, a null result is common [11,13,29,51–56]. However, interpretation of these results has been difficult due to methodological issues such as experimental design or the heterogeneity of participants. These factors were directly addressed in the design of the current study. Our visually-guided reaching task did sufficiently challenge the attentional resources of the participants and multiple methodological factors were controlled to achieve a homogenous sample of concussed participants, which led to no detectable behavioral differences as compared with control participants on the visually-guided reaching task.

## Limitations

While extensive efforts were made to ensure a strong experimental design, some limitations exist. Specifically, these findings are only applicable to young adult females, aged 18–28 who had a history of concussion and were asymptomatic by current standards. If an individual sustained her concussion in a competitive sports environment, the likelihood of entering a return-to-play protocol is high. The current guidelines for the return-to-play protocol incorporate both gradual increases in exercise and cognitive load/training [57]. Both exercise [58–60] and cognitive training, or neurorehabilitation [58], can aid in recovery of function after a traumatic brain injury via neural plasticity. Previous participation in a return-to-play protocol was not assessed in this study. Therefore, the influence of a return-to-play protocol on the results of this study is unknown.

The influence of individual motivation and competitiveness was not assessed in this study. Individuals may differ in their motivation and competitiveness when completing a task. Individuals high in competitiveness, or achievement motivation, will attempt to attain a better performance at a task whether measured against themselves or another group of individuals [61]. This relationship has been observed in both academic and sports settings [62] as well as in experimental tasks measuring reaction time when the difficulty of the task was varied [63,64]. Additionally, the effects of an individual’s motivation to ‘do well’ on a behavioral task or assessment has been investigated in previous studies of individuals with a history of concussion; however, the primary focus was centered on the assessments pre- and postinjury during the acute phase [65–67]. Simply put, the motivation of a previously concussed athlete during the postinjury assessment can lead to better performance on the evaluation so they can quickly return to their sport. Moreover, some evidence does exist for a participant’s motivation to influence task performance in individuals with a history of concussion in the long-term postinjury phase [68]. Therefore, some participants may have had higher achievement motivation than other participants or the previously concussed participants may have been motivated to perform better on the visually-guided reaching task to downplay the long-term effects of their injury.

One last potential limitation is a decrease in the statistical power of our follow-up comparisons due to our lower sample sizes after removing various participants as explained above, possibly accounting for our negative results. When comparing the effect sizes across the original analyses with the subsequent analyses, we did not see significant changes (See Supplementary Table 1 for an example of changes in the partial eta squared across two follow-up analyses with the greatest decrement in participant group sizes), thus the smaller group sizes did not appear to contribute to the lack of significant findings.

## Future perspective

As concussions continue to capture the attention of both the popular press and scientific community, the importance of investigating functional motor behaviors, such as visually-guided reaching, will continue to rise. Novel reaching tasks, such as the one designed for this study, have the potential to help researchers further understand the recovery of the brain postconcussion. Tasks designed to alter the cognitive load as well as to challenge the potentially diminished attentional resources in individuals with a remote history of concussion may lead to insights as to previously undetected deficits in brain function. Additionally, the visually-guided reaching task in this study should not be limited to postconcussion investigations. This task may prove beneficial in detecting cognitive or motor deficits in various populations such as older adults, stroke survivors, individuals diagnosed with Alzheimer’s disease or persons recovering from a concussion who are still symptomatic. The number of studies examining functional behaviors will continue to rise as the benefit of such investigations may be the steppingstones leading to developing interventions.

**Summary points**

- Mild traumatic brain injuries, or concussions, continue to be a major public health concern yet the number of studies investigating visually-guided motor behaviors in individuals with a remote history of concussions is limited.
- Females continue to be an underrepresented population in the concussion literature and thus the exclusive participants in this study.
- By altering the cognitive and motor demand required to successfully complete the visually-guided reaching task in this study, the goal of this study was to sufficiently challenge the participants as to reveal previously undetected performance deficits.
- The major finding of this study was that no significant differences existed between the two experimental groups after increasing both cognitive and motor demand of the visually-guided reaching task.
- Given the robust findings related to the experimental design of the visually-guided reaching task, the methods used in this study were sensitive enough to reveal any potential detectable difference between the two groups.
- The variables of age, sex, time since injury (greater than 6-month post injury), effects of caffeine and nicotine, sleep loss, sports participation, suspected concussions, multiple concussions and the effects of psychoactive drugs were all either controlled or considered during the analyses, yet still yielded a null result for group differences.
- The previously concussed, asymptomatic, female participants in this study performed equal to their nonconcussed control participants.
- Factors such as participation in a return-to-play protocol and the psychological attributes (i.e., competitiveness and motivation) of the participants were not assessed which could have influenced the results. Future studies should take these recommendations under consideration.

## References

[1] National Center for Injury Prevention and Control. Report to congress on mild traumatic brain injury in the United States: steps to prevent a serious public health problem (2003). https://stacks.cdc.gov/view/cdc/6544

[2] Bryan MA, Rowhani-Rahbar A, Comstock RD, Rivara F Sports- and recreation-related concussions in UW youth. Pediatrics 138(1), e20154635 (2016). 27325635 10.1542/peds.2015-4635

[3] Echlin PS, Tator CH, Cusimano MD A prospective study of physician-observed concussions during junior ice hockey: implications for incidence rates. Neurosurg. Focus 29(5), E4 (2010). 10.3171/2010.9.FOCUS10186 21039138

[4] McCrea M, Hammeke T, Olsen G, Leo P, Guskiewicz K Unreported concussion in high school football players: implications for prevention. Clin. J. Sport Med. 14(1), 13–17 (2004). 14712161 10.1097/00042752-200401000-00003

[5] Teasdale TW, Engberg A Duration of cognitive dysfunction after concussion, and cognitive dysfunction as a risk factor: a population study of young men. BMJ. 315(7108), 569–572 (1997). 9302952 10.1136/bmj.315.7108.569 PMC2127389

[6] Guskiewicz KM, Marshall SW, Bailes J Association between recurrent concussion and late-life cognitive impairment in retired professional football players. Neurosurgery 57(4), 719–726 (2005). 16239884 10.1093/neurosurgery/57.4.719

[7] Covassin T, Elbin R, Kontos A, Larson E Investigating baseline neurocognitive performance between male and female athletes with a history of multiple concussion. J. Neurol. Neurosurg. Psychiatry 81(6), 597–601 (2010). 20522868 10.1136/jnnp.2009.193797

[8] Covassin T, Moran R, Wilhelm K Concussion symptoms and neurocognitive performance of high school and college athletes who incur multiple concussions. Am. J. Sports Med. 41(12), 2885–2889 (2013). 23959963 10.1177/0363546513499230

[9] Iverson GL, Echemendia RJ, Lamarre AK, Brooks BL, Gaetz MB Possible lingering effects of multiple past concussions. Rehabil. Res. Pract. 2012, 316575 (2012). 22550590 10.1155/2012/316575 PMC3328154

[10] Collins MW, Grindel SH, Lovell MR Relationship between concussion and neuropsychological performance in college football players. JAMA 282(10), 964–970 (1999). 10485682 10.1001/jama.282.10.964

[11] Gardner A, Shores EA, Batchelor J Reduced processing speed in rugby union players reporting three or more previous concussions. Arch. Clin. Neuropsychol. 25(3), 174–181 (2010). 20202986 10.1093/arclin/acq007

[12] Shuttleworth-Rdwards AB, Radloff SE Compromised visuomotor processing speed in players of Rugby Union from school through to the national adult level. Arch. Clin. Neuropsychol. 23(5), 511–520 (2008). 18585890 10.1016/j.acn.2008.05.002

[13] Wall SE, Williams WH, Cartwright-Hatton S Neuropsychological dysfunction following repeat concussions in jockeys. J. Neurol. Neurosurg. Psychiatry 77(4), 518–520 (2006). 16543534 10.1136/jnnp.2004.061044 PMC2077488

[14] De Beaumont L, Henry LC, Gosselin N Long-term functional alterations in sports concussion. Neurosurg. Focus 33(6), E8, 1–7 (2012). 10.3171/2012.9.FOCUS12278 23199431

[15] Heitger MH, Jones RD, Dalrymple-Alford JC, Frampton CM, Ardagh MW, Anderson TJ Motor deficits and recovery during the first year following mild closed head injury. Brain Inj. 20(8), 807–824 (2006). 17060148 10.1080/02699050600676354

[16] Brown JA, Dalecki M, Hughes C, MacPherson AK, Sergio LE Cognitive-motor integration deficits in young adult athletes following concussion. BMC Sports Sci. Med. Rehabil. 7, 25 (2015). 26491541 10.1186/s13102-015-0019-4 PMC4612424

[17] Lavie N, De Fockert JW Contrasting effects of sensory limits and capacity limits in visual selective attention. Percept. Psychophys. 65(2), 202–212 (2003). 12713239 10.3758/bf03194795

[18] Fougnie D, Marois R Distinct capacity limits for attention and working memory: evidence from attentive tracking and visual working memory paradigms. Psychol. Sci. 17(6), 526–534 (2006). 16771804 10.1111/j.1467-9280.2006.01739.x

[19] Hyndman D, Ashburn A People with stroke living in the community: attention deficits, balance, ADL ability and falls. Disabil. Rehabil. 25(15), 817–822 (2003). 12851091 10.1080/0963828031000122221

[20] Tippett WJ, Sergio LE Visuomotor integration is impaired in early stage Alzheimer’s disease. Brain Res. 1102(1), 92–102 (2006). 16797495 10.1016/j.brainres.2006.04.049

[21] Gagnon I, Swaine B, Friedman D, Forget R Visuomotor response time in children with a mild traumatic brain injury. J. Head Trauma Rehabil. 19(5), 391–404 (2004). 15597030 10.1097/00001199-200409000-00004

[22] Heitger MH, Anderson TJ, Jones RD, Dalrymple-Alford JC, Frampton CM, Ardagh MW Eye movement and visuomotor arm movement deficits following mild closed head injury. Brain 127(3), 575–590 (2004). 14736751 10.1093/brain/awh066

[23] Catena RD, Van Donkelaar P, Halterman CI, Chou LS Spatial orientation of attention and obstacle avoidance following concussion. Exp. Brain Res. 194(1), 67–77 (2009). 19082819 10.1007/s00221-008-1669-1

[24] Baker CS, Cinelli ME Visuomotor deficits during locomotion in previously concussed athletes 30 or more days following return to play. Physiol. Rep. 2(12), e12252 (2014). 25539832 10.14814/phy2.12252 PMC4332226

[25] Locklin J, Bunn L, Roy E, Danckert J Measuring deficits in visually guided action post-concussion. Sports Med. 40(3), 183–187 (2010). 20199118 10.2165/11319440-000000000-00000

[26] Covassin T, Elbin RJ, Harris W, Parker T, Kontos A The role of age and sex in symptoms, neurocognitive performance, and postural stability in athletes after concussion. Am. J. Sports Med. 40(6), 1303–1312 (2012). 22539534 10.1177/0363546512444554

[27] Collins MW, Lovell MR, Iverson GL, Cantu RC, Maroon JC, Field M Cumulative effects of concussion in high school athletes. Neurosurgery 51(5), 1175–1181 (2002). 12383362 10.1097/00006123-200211000-00011

[28] Belanger HG, Spiegel E, Vanderploeg RD Neuropsychological performance following a history of multiple self-reported concussions: a meta-analysis. J. Int. Neuropsychol. Soc. 16(2), 262–267 (2010). 20003581 10.1017/S1355617709991287

[29] Iverson GL, Brooks BL, Lovell MR, Collins MW No cumulative effects for one or two previous concussions. Br. J. Sports Med. 40(1), 72–75 (2006). 16371496 10.1136/bjsm.2005.020651 PMC2491929

[30] Covassin T, Schatz P, Swanik CB Sex differences in neuropsychological function and postconcussion symptoms of concussed collegiate athletes. Neurosurgery 61(2), 345–351 (2007). 17762747 10.1227/01.NEU.0000279972.95060.CB

[31] Broglio SP, Puetz TW The effect of sport concussion on neurocognitive function, self-report symptoms and postural control: a meta-analysis. Sports Med. 38(1), 53–67 (2008). 18081367 10.2165/00007256-200838010-00005

[32] Faul F, Erdfelder E, Lang A-G, Buchner A G*Power 3: a flexible statistical power analysis program for the social, behavioral and biomedical sciences. Behav. Res. Methods 39(2), 175–191 (2007). 17695343 10.3758/bf03193146

[33] Hindmarch I Psychomotor function and psychoactive drugs. Br. J. Clin. Pharmac. 58(7), S720–S740 (2004). 10.1111/j.1365-2125.2004.02279.x PMC1884665 15595961

[34] Smith A, Sturgess W, Gallagher J Effects of a low dose of caffeine given in different drinks on mood and performance. Hum. Psychopharmacol. Clin. Exp. 14(7), 473–482 (1999).

[35] Hallowell B Strategic design of protocols to evaluate vision in research on aphasia and related disorders. Aphasiology 22(6), 600–617 (2008).

[36] Labotz M, Martin MR, Kimura IF, Hetzler RK, Nichols AW A comparison of a preparticipation evaluation history form and a symptom-based concussion survey in the identification of previous head injury in collegiate athletes. Clin. J. Sport Med. 15(2), 73–78 (2005). 15782050 10.1097/01.jsm.0000157649.99867.fc

[37] Guskiewicz KM, Register-Mihalik J, McCrory P Evidence-based approach to revising the SCAT2: introducing the SCAT3. Br. J. Sports Med. 47(5), 289–293 (2013). 23479486 10.1136/bjsports-2013-092225

[38] Durlach PJ The effects of a low dose of caffeine on cognitive performance. Psychopharmacology 140(1), 116–119 (1998). 9862410 10.1007/s002130050746

[39] Stocker RP, Khan H, Henry L, Germain A Effects of sleep loss on subjective complaints and objective neurocognitive performance as measured by the immediate post-concussion assessment and cognitive testing. Arch. Clin. Neuropsychol. 32(3), 349–368 (2017). 28431034 10.1093/arclin/acx003

[40] Sabbe B, Van Hoof J, Hulstijn W, Zitman F Changes in fine motor retardation in depressed patients treated with fluoxetine. J. Affect. Disord. 40(3), 149–157 (1996). 8897114 10.1016/0165-0327(96)00052-3

[41] Paul MA, Gray G, Lange M The impact of sertraline on psychomotor performance. Aviat. Space Environ. Med. 73(10), 964–970 (2002). 12398257

[42] Tegner Y, Lysholm J Rating systems in the evaluation of knee ligament injuries. Clin. Orthop. Relat. Res. 198, 43–49 (1985). 4028566

[43] Oldfield RC The assessment and analysis of handedness: the Edinburgh inventory. Neuropsychologia 9(1), 97–113 (1971). 5146491 10.1016/0028-3932(71)90067-4

[44] Holm S A simple sequentially rejective multiple test procedure. Scand. J. Stat. 6(2), 65–70 (1979).

[45] Huddleston WE, Aleksandrowicz MS, Yufa A, Knurr CR, Lytle JR, Puissant MM Attentional resource allocation during a cued saccade task. Acta Psychol. 144(1), 112–120 (2013). 10.1016/j.actpsy.2013.05.006 23792667

[46] Beurskens R, Steinberg F, Antoniewicz F, Wolff W, Granacher U Neural correlates of dual-task walking: effects of cognitive versus motor interference in young adults. Neural Plast. 8032180 (2016). 27200192 10.1155/2016/8032180 PMC4855015

[47] Kelly AM, Uddin LQ, Biswal BB, Castellanos FX, Milham MP Competition between functional brain networks mediates behavioral variability. Neuroimage 39(1), 527–537 (2008). 17919929 10.1016/j.neuroimage.2007.08.008

[48] Dougan BK, Horswill MS, Geffen GM Athletes’ age, sex, and years of education moderate the acute neuropsychological impact of sports-related concussion: a meta-analysis. J. Int. Neuropsychol. Soc. 20(1), 64–80 (2014). 23375058 10.1017/S1355617712001464

[49] Belanger HG, Curtiss G, Demery JA, Lebowitz BK, Vanderploeg RD Factors moderating neuropsychological outcomes following mild traumatic brain injury: a meta-analysis. J. Int. Neuropsychol. Soc. 11(3), 215–227 (2005). 15892898 10.1017/S1355617705050277

[50] Belanger HG, Vanderploeg RD The neuropsychological impact of sports-related concussion: a meta-analysis. J. Int. Neuropsychol. Soc. 11(4), 345–357 (2005). 16209414 10.1017/s1355617705050411

[51] De Beaumont L, Theoret H, Mongeon D Brain function decline in healthy retired athletes who sustained their last sports concussion in early adulthood. Brain 132(3), 695–708 (2009). 19176544 10.1093/brain/awn347

[52] Baillargeon A, Lassonde M, Leclerc S, Ellemberg D Neuropsychological and neurophysiological assessment of sport concussion in children, adolescents and adults. Brain Inj. 26(3), 211–220 (2012). 22372409 10.3109/02699052.2012.654590

[53] Broglio SP, Pontifex MB, O’Connor P, Hillman CH The persistent effects of concussion on neuroelectric indices of attention. J. Neurotrauma 26(9), 1463–1470 (2009). 19331519 10.1089/neu.2008.0766

[54] Guskiewicz KM, Marshall SW, Broglio SP, Cantu RC, Kirkendall DT No evidence of impaired neurocognitive performance in collegiate soccer players. Am. J. Sports Med. 30(2), 157–162 (2002). 11912081 10.1177/03635465020300020201

[55] Theriault M, De Beaumont L, Tremblay S, Lassonde M, Jolicoeur P Cumulative effects of concussions in athletes revealed by electrophysiological abnormalities on visual working memory. J. Clin. Exp. Neuropsychol. 33(1), 30–41 (2011). 20480425 10.1080/13803391003772873

[56] Broglio SP, Ferrara MS, Piland SG, Anderson RB Concussion history is not a predictor of computerised neurocognitive performance. Br. J. Sports Med. 40(9), 802–805 (2006). 16929049 10.1136/bjsm.2006.028019 PMC2564398

[57] McCrory P, Meeuwisse W, Aubry M Consensus statement on concussion in sport--the 4th International Conference on Concussion in Sport held in Zurich, November 2012. J. Sci. Med. Sport 16(3), 178–189 (2013). 23541595 10.1016/j.jsams.2013.02.009

[58] Greenwood RS, Parent JM Damage control: the influence of environment on recovery from brain injury. Neurology 59(9), 1302–1303 (2002). 12427874 10.1212/wnl.59.9.1302

[59] Berlucchi G Brain plasticity and cognitive neurorehabilitation. Neuropsychol. Rehabil. 21(5), 560–578 (2011). 21563013 10.1080/09602011.2011.573255

[60] Van Praag H Exercise and the brain: something to chew on. Trends Neurosci. 32(5), 283–290 (2009). 19349082 10.1016/j.tins.2008.12.007 PMC2680508

[61] Nicholls JG Achievement motivation: conceptions of ability, subjective experience, task choice and performance. Psychol. Rev. 91(5), 328–346 (1984).

[62] Duda JL, Nicholls JG Dimensions of achievement motivation in schoowork and sport. J. Educ. Psychol. 84(3), 290–299 (1992).

[63] Capa RL, Audiffren M, Ragot S The effects of achievement motivation, task difficulty, and goal difficulty on physiological, behavioral, and subjective effort. Psychophysiology 45(5), 859–868 (2008). 18627535 10.1111/j.1469-8986.2008.00675.x

[64] Wamkel LM Competition in motor performance: an experimental analysis of motivational components. J. Exp. Soc. Psychol. 8(5), 427–437 (1972).

[65] Rabinowitz AR, Arnett PA Intraindividual cognitive variability before and after sports-related concussion. Neuropsychology 27(4), 481–490 (2013). 23876120 10.1037/a0033023

[66] Echemendia RJ, Herring S, Bailes J Who should conduct and interpret the neuropsychological assessment in sports-related concussion? Br. J. Sports Med. 43(Suppl. 1), i32–i35 (2009). 19433423 10.1136/bjsm.2009.058164

[67] Bailey CM, Echemendia RJ, Arnett PA The impact of motivation on neuropsychological performance in sports-related mild traumatic brain injury. J. Int. Neuropsychol. Soc. 12(4), 475–484 (2006). 16981599 10.1017/s1355617706060619

[68] De Beaumont L, Mongeon D, Tremblay S Persistent motor system abnormalities in formerly concussed athletes. J. Athl. Train. 46(3), 234–240 (2011). 21669091 10.4085/1062-6050-46.3.234 PMC3419550

